# Spore surface proteins of *Brevibacillus laterosporus* are involved in insect pathogenesis

**DOI:** 10.1038/srep43805

**Published:** 2017-03-03

**Authors:** Maria Giovanna Marche, Maria Elena Mura, Giovanni Falchi, Luca Ruiu

**Affiliations:** 1 Dipartimento di Agraria, University of Sassari, Sassari, 07100, Italy

## Abstract

Outer spore envelope proteins of pathogenic bacteria often present specific virulence factors and tools to evade the defence system of their hosts. *Brevibacillus laterosporus,* a pathogen of invertebrates and an antimicrobial-producing species, is characterised by a unique spore coat and canoe-shaped parasporal body (SC-CSPB) complex surrounding the core spore. In the present study, we identified and characterised major proteins of the SC-CSPB complex of *B. laterosporus*, and we investigated their entomopathogenic role. Employing a proteomic approach and a *B. laterosporus*-house fly study model, we found four highly conserved proteins (ExsC, CHRD, CpbA and CpbB) that function as insect virulence factors. CpbA was associated with a significantly higher mortality of flies and greater relative gene expression levels during sporulation, compared to the other SC-CSPB proteins. Taken together, we suggest that spore surface proteins are a part of a complex set of toxins and virulence factors that *B. laterosporus* employs in its pathogenicity against flies.

The primary function of bacterial cell envelopes is to provide structural integrity and protection to the protoplast. Despite significant differences among diverse species, their composition normally includes envelope polymers and surface layer proteins that bacteria typically use to interact with the external environment^1^. In endospore forming species (i.e. *Bacillus* spp., *Clostridium* spp.), the spore is encased in a protein coat that can be additionally surrounded by outermost structures like the exosporium that protects the spores and interacts with the environment^2^. Surface proteins are a main feature of pathogenic bacteria that have developed specific virulence factors and approaches to evade the defence system of their hosts^3^. Better understanding of the cell and spore surface layer organisation and function are providing new insights for biotechnology and medical applications^4^.

Similarly, pathogenic bacteria have evolved a wide variety of toxicity mechanisms and strategies to invade the body of insects, overcoming their immune defences^5^,^6^. A plethora of molecules produced by different species, lead to a multiplicity of pathogen-host interactions. Among these are the insecticidal crystal toxins (Cry) typical of *Bacillus thuringiensis* and *Lysinibacillus sphaericus*, and the insecticidal toxin complexes of gammaproteobacteria like the entomopathogenic nematode symbionts *Photorhabdus* and *Xenorhabdus* species (Tc), *Serratia* species (Sep), and *Yersinia entomophaga* (Yen-Tc)^7^. In certain cases, insect virulence factors are localised on the outermost spore layers as demonstrated in the *Bacillus cereus* group^8^,^9^,^10^.

*Brevibacillus laterosporus,* a pathogen of invertebrates and an antimicrobial species, is morphologically characterised by a typical spore surrounded by a firmly attached canoe-shaped parasporal body (CSPB). The biocontrol potential of this bacterial species in agriculture is not limited to invertebrate pests (insects in different orders, nematodes and mollusks) but also includes phytopathogenic bacteria and fungi^11^. This broad-spectrum of activity is associated with a wide variety of molecules, including proteins and antibiotics, it produces. Whilst there are significant differences among strains in terms of virulence, the results of the recent whole genome sequencing and annotation of reference strain LMG 15441 revealed a conserved potential of this species to produce several polyketides, nonribosomal peptides, and toxins^12^. Pathogenicity against dipterans, including mosquitoes and flies, has been associated with the ingestion of sporulated cultures with or without parasporal bodies^13^,^14^,^15^,^16^. Despite the possible implication of spore surface proteins in the toxic interaction with the midgut of house flies, the virulence factors acting against these insects have not been identified^17^,^18^.

In the present study, we identified and characterised the main surface proteins of *B. laterosporus* spores and we tested the hypothesis that they could be involved in its pathogenicity. Employing a *B. laterosporus*-house fly study model, we found that some of the proteins associated with the spore coat, exosporium and CSPB complex represent putative virulence factors acting against insects. Furthermore, we showed that as these proteins are progressively synthesised during bacterial growth, a proportional increase in the insecticidal activity of this bacterium is observed, being fully toxic when the spore envelopes are completely formed.

## Results

### Virulence increases during bacterial growth reaching a maximum at sporulation

**Figure 1:**
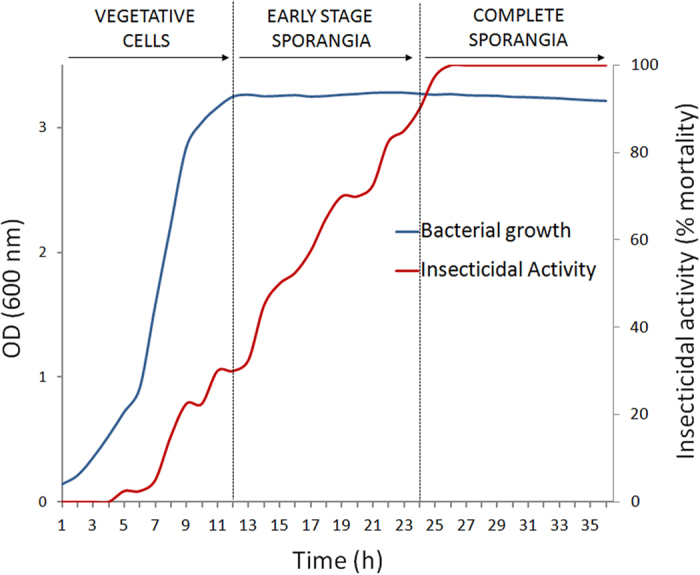
Fly mortality rates associated with progressive bacterial growth. The average mortality rates and bacterial growth are plotted against time.

The majority of studies reporting the insecticidal action of *B. laterosporus* strains against mosquitoes and flies have been conducted employing sporulated cultures obtained with different preparation methods that may lead to bacterial suspensions consisting of old vegetative cells, unlysed sporangia and free spores mixed in varying proportions^19^. In order to evaluate the actual virulence of consecutive stages of growth, we conducted preliminary time-course experiments based on synchronised cultures of the well documented house fly pathogen *B. laterosporus* strain UNISS 18^17^. The relationship between bacterial growth and insecticidal activity is shown in Fig. 1. Significant lethal effects were recorded at the beginning of the sporulation phase and increased progressively reaching a maximum when spores were completely formed inside sporangia (F_36,111_ = 208.06; P < 0.0001). Virulence was maintained when free spores were released by lysed sporangia.

**Figure 2:**
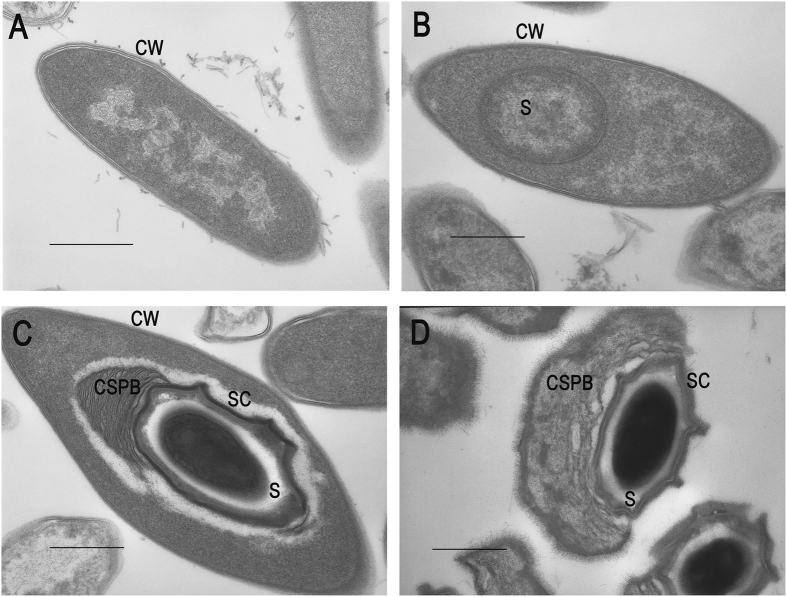
Representative transmission electron microscopy (TEM) micrographs of different *Brevibacillus laterosporus* growth stages. Vegetative cell (**A**). Early-stage swollen sporangium (**B**). Sporangium including completely formed spore (**C**). Free spore (**D**). CW: cell wall, S: spore; SC: spore coat, CSPB: canoe-shaped parasporal body. Bar: 500 nm.

Remarkable, parallel to the increase in virulence was the progressive synthesis of the bacterial spore coat and canoe-shaped parasporal body (SC-CSPB) complex (Fig. 2).

No significant toxicity was associated with negative controls based on saccharose solutions or suspensions of *B. thuringiensis* HD1 cultures.

### Isolation and identification of SC-CSPB proteins

The main hypothesis in this study is that SC-CSPB surface proteins are involved in the pathogenic action displayed in the insect gut immediately after ingestion. For this reason, specific investigations were conducted to identify and isolate major proteins of the SC-CSPB complex.

**Figure 3:**
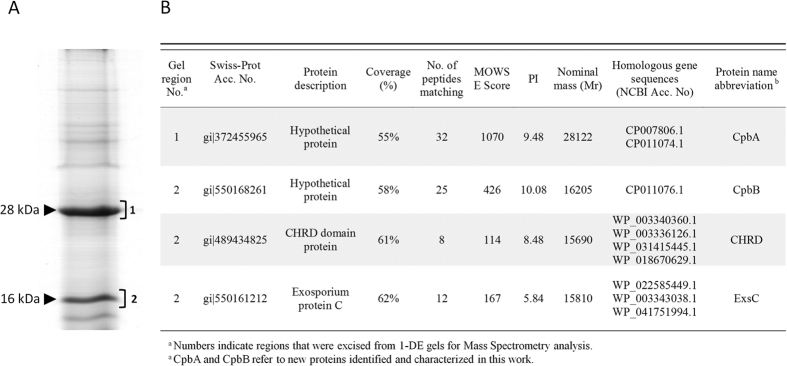
Major proteins from the *Brevibacillus laterosporus* SC-CSPB complex. (**A**) Protein pattern of alkali-extracted proteins from the SC-CSPB complex. (**B**) Mass spectrometry identification of major proteins.

The 1-DE profile of surface proteins, specifically extracted by alkali and a reducing agent, showed two major bands corresponding to an apparent molecular weight around 28 and 16 kDa, respectively. Four different proteins were detected in LC-MS/MS analysis of the main bands: two hypothetical proteins, that we designated CpbA and CpbB (Cpb, spore coat-parasporal body complex), a Cysteine*-* and histidine-rich domain protein (CHRD), and an Exosporium protein C (ExsC) (Fig. 3).

### Cloning, sequencing and expression of *SC-CSPB* genes

*CpbA, cpbB, chrd* and *exsC* genes of *B. laterosporus* UNISS 18 were successfully amplified with primers designed on homologous sequences available on NCBI (National Centre for Biotechnology Information, Bethesda, Maryland) database, and the PCR fragments were ligated into pGEX-3X vector used to transform *E. coli* DH5α. Recombinant plasmids from generated clones were used for gene sequencing. DNA sequences of these genes were deposited in the GenBank database under the following accession numbers: KY124461 (*cpbA*), KY124462 (*cpbB*), KY124463 (*chrd*), and KY124464 (*exsC*). An alignment of available genome sequences in the NCBI database showed that these genes are highly conserved in *B. laterosporus* with 99% identity. While *cpbA, CHRD* and *exsC* genes are present in all the available *B. laterosporus* genomes (strains LMG 15441, GI-9, DSM 25, B9, PE36), *cpbB* gene was found only in some of them (GI-9, B9, PE36), in addition to UNISS 18.

**Figure 4:**
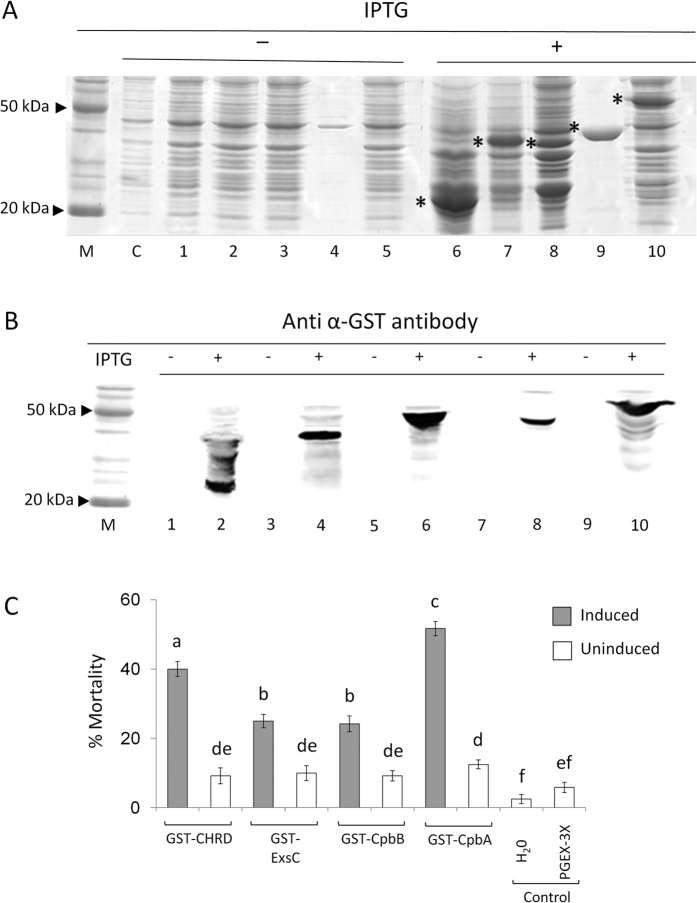
Protein expression patterns and virulence of transformed *E. coli* BL21 cells expressing *B. laterosporus* fusion proteins. (**A**) SDS-PAGE showing the protein profile of uninduced (−) or IPTG-induced (+) *E. coli* BL21 cells transformed with pGEX-3X fusion constructs including different SC-CSPB genes cloned at the C-terminal of the GST gene; M, premixed protein marker (Promega); (**C**), BL21; 1, pGEX-3X; 2, pGEX-*exsC*; 3, pGEX-*CHRD*; 4, pGEX-*cpbB*, 5, pGEX-*cpbA* ; 6, pGEX-3X; 7, pGEX-*exsC*; 8, pGEX-*CHRD*; 9, pGEX-*cpbB*, 10, pGEX-*cpbA* ; asterisk (*) indicates the expression of GST-tagged proteins; (**B**) Western blot using anti α-GST antibody on protein extracts from uninduced (−) and IPTG-induced (+) *E. coli* BL21 cells transformed with pGEX-3X (1, 2), pGEX-*exsC* (3, 4), pGEX-*CHRD* (5, 6), pGEX-*cpbB* (7, 8), pGEX-*cpbA* (9, 10); (**C**) Mortality change (mean ± SEM) of house flies treated lysates of induced vs uninduced *E. coli* BL21 cells (protein concentration: 2 mg/ml) expressing SC-CSPB proteins; different letters above the bars indicate significantly different means (ANOVA, followed by LSD test, p < 0.001).

**Figure 5:**
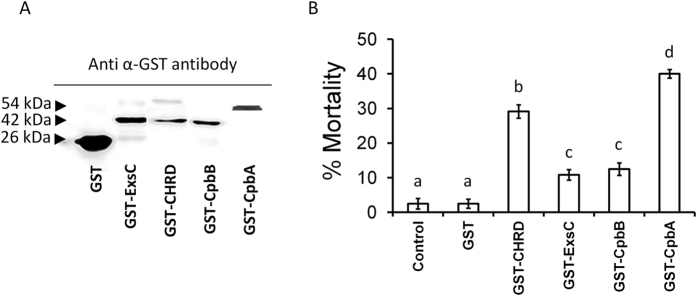
Mortality of house flies exposed to SC-CSPB fusion proteins. (**A**) Western blot using anti α-GST antibody on SC-CSPB fusion proteins purified from lysates of IPTG-induced *E. coli* BL21 cells transformed with pGEX-3X fusion constructs; (**B**) Mortality (mean ± SEM) of house flies treated with SC-CSPB fusion proteins (1 mg/ml of diet); different letters above the bars indicate significantly different means (ANOVA, followed by LSD test, p < 0.001).

Genes *cpbA, cpbB, chrd* and *exsC* cloned in pGEX-3X expression vector at the C-terminal end of the glutathione S-transferase gene (GST) were used to transform *E. coli* BL21. Recombinant proteins were produced in the cell following induction of gene expression with IPTG, as demonstrated by the 1-DE protein profile of cell lysates followed by Western Blot analysis (Fig. 4A and B). As expected, fusion protein bands representing ExsC (42 kDa), CHRD (42 kDa), CpbA (54 kDa), and CpbB (42 kDa), presumably comprising GST (26 KDa), were observed only on 1-DE gels for *E. coli* cells induced by IPTG, in comparison with uninduced cultures. Slight differences between the apparent and expected molecular weight of fusion proteins in Fig. 4A and B are attributable to their concentration and proportion in the protein mixtures extracted from diverse *E. coli* cultures. Consistently, sequencing confirmed the integrity of the protein gene sequences in the pGEX-3X expression vector, and we do not have any indication of post-translational modification of these recombinant proteins in *E. coli*. Accordingly, all GST-tagged proteins were successfully purified from bacterial cell lysates by affinity chromatography, and correspondence between apparent and expected protein size was observed, as confirmed by 1-DE and Western Blot analysis (Fig. 5A).

### Insect bioassays. SC-CSPB proteins as virulence factors

Suspensions of transformed *E. coli* BL21 cells expressing CpbA, CpbB, CHRD and ExsC fusion proteins, after being induced by IPTG, were lysed and assayed on house fly adults to assess their toxicity. Cells expressing GST-tagged proteins showed significantly higher fly mortality compared with uninduced cells (F_9,110_ = 73.06, P < 0.001), with cells bearing the *chrd* and *cpbA* genes showing the highest virulence. No significant mortality was associated with controls (Fig. 4C).

Significant fly mortality was also shown by all purified fusion proteins, though, at varying degrees: 40% for CpbA, 30% for CHRD, 12% for CpbB, and 10% for ExsC (F_4,67_ = 124.65, P < 0.001) (Fig. 5B). GST alone did not show any lethal effect, compared to the control.

### Time-course expression of SC-CSPB genes during bacterial growth in culture

**Figure 6:**
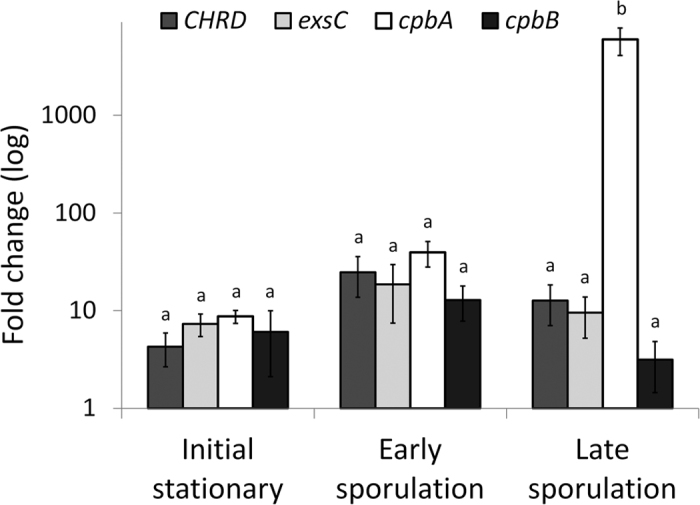
Time course expression of SC-CSPB complex genes. Relative expression profiles of *exsC, CHRD, cpbA, and cpbB* genes (mean ± SEM), detected using RT-qPCR on synchronized *B. laterosporus* cultures in correspondence of progressive stages of growth. Ct values were normalized by using 16S rRNA as reference gene and fold changes were calculated relative to the average expression during the exponential vegetative phase by using the 2^−ΔΔCt^ method. Different letters above the bars indicate significantly different means (ANOVA (PROC MIXED), followed by LSMEANS comparison (adjust = Turkey), p < 0.001).

To support the hypothesis that the increase in virulence of *B. laterosporus* during sporulation is related to the augmented synthesis of putative virulence factors, the expression profiles of *cpbA, cpbB, chrd* and *exsC* genes were detected using RT-qPCR on synchronised *B. laterosporus* cultures at various stages of growth: initial stationary (12 h), early sporulation (18 h) and late sporulation (24 h) phases. As shown in Fig. 6, all target genes were expressed during the stationary phase and their expression level increased during sporulation (F_32,64_ = 9.22, P = 0.0002). A slight-though-not-significant increase in *cpbB, chrd* and *exsC* transcript levels in early sporulation was followed by a later decrease; for *cpbA,* we observed a significant, more than a hundred-fold, increment during late sporulation.

## Discussion

Among the variety of toxins that pathogenic bacteria employ as virulence factors during infectious diseases^20^, the components of the outer spore envelope layers normally play a main role in the pathogen-host interactions, especially during the initial infection stages^21^. The present study identified major proteins of the spore coat and canoe-shaped parasporal body (SC-CSPB) complex, including the exposporium, surrounding the core spore of *B. laterosporus* UNISS 18, and suggests their implication in the pathogenic action against insects.

Several studies reported that the insecticidal action of different *B. laterosporus* strains was observed when insects were exposed to bacterial cultures in the stationary or sporulation phase of growth. During this time, a bacterial mother cell normally produces the coat proteins that are progressively layered onto the spore surface^22^. Such process must be particularly energy consuming for a microorganism like *B. laterosporus* that has to build a SC-CSPB complex even larger than the core spore itself. Our time-course experiments based on synchronised cultures confirm the toxicity of late stationary phase cells and the achievement of full insecticidal potential when the spores are completely formed within the sporangium. According to previous studies indicating the spore as the main active ingredient of *B. laterosporus-*based insecticidal preparations against the house fly^17^, our results imply that the spore holds specific toxic factors against this insect species. In fact, the death of flies ingesting spores, normally occurs before spore germination^18^. It is well documented that the variations in outer spore envelope composition of different bacterial species reflect adaptations to specific ecological niches^1^. It can be inferred that *B. laterosporus* must have developed its own tools to overcome host defence mechanisms.

Employing a proteomic approach, we have successfully isolated and identified four major proteins of the SC-CSPB complex that are highly conserved among members of this bacterial species: CpbA, CpbB, CHRD and ExsC. Interestingly, the genes encoding these proteins are shared with other *B. laterosporus* genomes so far sequenced^12^,^23^,^24^. To date, no function has been associated with the two hypothetical proteins that we designated CpbA and CpbB, where Cpb stands for spore coat-parasporal body complex. In addition, no information on their actual expression and localisation in the bacterial cell was available. In our study, we found them to be major components of the SC-CSPB complex. Consistently, it was previously suggested that approximately 25 per cent of the weight of the canoe-coat structure is extractable protein and that one or two main proteins are included in the canoe^25^. Transmission electron microscopy micrographs clearly show a lamellar structure that is firmly attached and continuous with the spore coat (Fig. 2). The construction of this structure begins in the late stationary phase and progresses during sporulation, which is corroborated by a general increased expression of the SC-CSPB complex genes.

Several external bacterial spore envelope proteins are normally found in members of the order Bacillales, as shown in the studies on different *Bacillus cereus* group species^21^. Our approach led to the isolation of CHRD and ExsC homologues, but we cannot exclude that other *B. laterosporus* proteins from the spore coat or the exosporium, may have similar or higher importance in the pathogenic process. Microbial CHRD domains are homologues to a protein motif identified in chordin, an inhibitor of bone morphogenetic proteins. This domain has an immunoglobulin-like β-barrel structure, but after its discovery in bacteria, no clear function has been predicted^26^. Given its key role in dorsalisation of the early vertebrate embryonic tissues^27^, it would be of great interest to investigate its possible implication in the lateral development of the canoe-shaped parasporal body of *B. laterosporus*. While this aspect would need to be specifically confirmed for *B. laterosporus*, a recent study suggested a critical role of the 15.4 kDa basic spore protein ExsM, corresponding to the CHRD domain, in the formation, size and shape of the *B. cereus* ATCC 4342 exosporium^28^. The involvement of the CHRD domain in the regulation of other biological pathways has also been proven^29^, but no information on its potential implication in the pathogenic action of bacteria against insects are available. Similarly, no specific association of spore protein ExsC homologues with bacterial virulence has been reported, and such role in *B. laterosporus* is not yet known. However, studies on species in the *B. cereus* group revealed the presence of *exsC* gene homologues encoding for structural protein components of *B. cereus* and *B. anthracis* exosporium, that would interact with host cells during infection, playing a significant role in the pathogenic process^30^. ExsC protein can also be found as a component of the paracrystalline basal layer of the *B. thuringiensis* exosporium^31^.

When house flies were exposed to lysates of transformed *E. coli* BL21 cells expressing either CpbA, CpbB, CHRD or ExsC fusion proteins, a significant increase in mortality was observed in comparison with uninduced cell lysates. Such mortality increase was also detected after insects ingested a diet containing purified fusion proteins. However, lethal effects were mild in comparison with the full toxicity that can usually be observed when flies ingest *B. laterosporus* spores, which would suggest that these proteins, rather than being actual insecticidal toxins, may represent supplementary virulence factors contributing to enhance bacterial virulence potential. However, we cannot exclude that some of these SC-CSPB proteins may lose most of their insecticidal potential when applied alone, rather than in combination with other toxins or with the whole spore. Future experiments involving combinations of spore surface proteins in different proportions will clarify if such synergisms are involved in the insecticidal action. This is especially true if we imagine entomopathogenesis as a complex process involving the contribution of several factors^32^.

Concerning CpbA protein, a significantly higher mortality of flies and a greater relative gene expression level during sporulation were observed compared to other SC-CSPB proteins. Therefore, we can confirm that this protein is likely to contribute significantly to pathogenicity. Since this protein has a significantly higher molecular mass compared to the other three, we cannot exclude that further differences may arise in assays employing these proteins in a similar molar concentration. Previous studies documented the ultrastructural changes caused by *B. laterosporus* UNISS 18 spores to the midgut epithelium of flies^18^, thus supporting a toxin mediated process reminiscent of the disruptive action induced by diverse bacterial insecticidal proteins^6^,^7^. Accordingly, SC-CSPB proteins might be directly involved in the interaction with epithelial cells. However, specific experiments are required to support such a claim. Rather than having a direct insecticidal action, these proteins might be involved in key interaction events inside the insect gut environment during pathogenesis. Their role would become negligible, when spore germination is followed by the release of more potent virulence factors of *B. laterosporus* like polyketides, nonribosomal peptides, chitinases, various proteases and toxins^12^,^33^,^34^.

On the other hand, the spore surface layers, including the canoe-shaped parasporal body, are supposed to provide both mechanical and chemical resistance to the spore core, thus hindering lysozyme action and the other immune-related defence mechanisms encountered during the passage through the house fly’s intestinal tract^18^, and enabling the spore to maintain its pathogenic potential^22^. According to this scenario, an abundant expression of the spore coat genes would be critical for a proper formation of this envelope^35^.

Because most of the SC-CSPB genes are shared with other *B. laterosporus* strains showing insecticidal potential against dipterans (i.e., LMG 15441, DSM 25)^14^,^19^, a conserved role of SC-CSPB proteins in pathogenesis is expected. However, differences among strains may derive from different gene expression levels.

Altogether, our results suggest that the proteins we isolated and identified in this study can be considered part of a more complex set of toxins and virulence factors involved in the insecticidal action of *B. laterosporus*. Future studies will clarify how these proteins are important to this action, whether they interact directly with the host or they just assist spores to carry out their pathogenic action.

## Methods

### Bacterial strains and growth conditions

*B. laterosporus* strain UNISS 18 (=NCIMB 41419), known to have insecticidal action against diptera was used in the present study^17^. Bacteria were routinely grown at 30 °C in conical flasks containing Luria–Bertani (LB) broth with shaking at 180 rpm. To obtain synchronised cultures, methods described in Ruiu *et al*.^17^ were followed. Briefly, a pre-culture in LB broth, started with a heat-activated spore suspension (1 ml), was used to inoculate a second LB culture of which an aliquot (25 ml) was used to inoculate the sporulation medium T3^36^ where synchronised bacterial growth, up to complete sporulation, was observed under phase microscopy. Specific T3 cultures (500 ml) were grown in three replicates to measure optical density (OD) at 600 nm every hour during growth. In different experiments, aliquots (50 ml) of the cultures at different time intervals were harvested by centrifugation at 5000 rpm for 20 min at 4 °C to recover pure suspensions of all bacterial phases of growth (exponential vegetative cells, stationary vegetative cells, sporangia, free spores) that were used in bioassays, for analysis, or stored at −80 °C until further use.

Suspensions of the crystal forming *Bacillus thuringiensis* strain HD1 were grown under the same conditions to be routinely used as negative controls in fly bioassays.

*Escherichia coli* DH5 α was used as cloning strain. As known, it is restriction-defective and has mutations in *relA1* and *recA1* genes, to improve stability and quality of recombinant plasmids^37^.

Chemically competent cells of *E. coli* BL21(DE3) (Novagen) were used for further transformation and protein expression. This *E. coli* strain is deficient in Lon and OmpT proteases and contain the T7 RNA polymerase gene, expressed upon addition of isopropyl-1-thio-β-D-galactopyranoside (IPTG).

*E. coli* was routinely grown at 37 °C on LB agar plate or LB broth with shaking at 200 rpm.

All cell and spore counts were performed using a Neubauer chamber (Brand Germany).

### Transmission electron microscopy

Bacteria were prepared for observations under transmission electron microscopy following general methods of ref. 38 with several adaptations. Briefly, cells and spores from harvested cultures were fixed in 4% glutaraldehyde and 4% paraformaldehyde in cacodylate buffer (0.05 M, pH 7.2) at 4 °C for 24 h, then washed in cacodylate buffer, suspended in low-melting point agarose that, after solidification, was trimmed into small pieces with a scalpel.

Agarose pieces with cell pellets were post-fixed in 1% osmium tetroxide for 1 h at 4 °C, followed by washing twice in the same buffer, before being dehydrated through a progressive ethanol gradient until absolute ethanol, and embedded in Epon-Araldite. Ultra-thin sections (about 70 nm thickness) were cut using a L.K.B. “Nova” ultramicrotome, mounted on grids and stained with uranyl acetate and lead citrate. Observations were successively conducted under a Zeiss EM 109 transmission electron microscope. Representative images were selected and micrographed.

### 1-DE separation of spore surface proteins

Synchronised cultures were used for surface proteins extraction and separation from spores.

Proteins from the SC-CSPB complex were extracted according to Fits-James and Young^25^ with few adaptations^17^. One millilitre of a spore suspension was harvested by centrifugation at 13,000 rpm for 10 min, and the pellet resuspended in 1 ml 0.1N NaOH – 1% thioglycolic acid. The suspension was titrated to pH 11.5 by adding 1 M NaOH and incubated at 30 °C for 30 min before being centrifuged at 13,000 rpm for 10 min. The supernatant was dialysed at 4 °C against water using 3,500 MWCO SnakeSkin^TM^ Pleated Dialysis tubing (Cole-Parmer Instrument Company, UK). Water was changed three times after 1 h, 2 h and overnight, and then the supernatant was collected for analysis. Protein concentration was routinely determined using the Folin phenol reagent^39^ and bovine serum albumin (Sigma) as a standard.

Protein samples were mixed with Laemmli buffer^40^, boiled for 5 min and run in a 10% or 15% SDS-PAGE gel using a Mini-Protean electrophoresis system (BioRad Laboratories Inc., USA). Gels were stained with Coomassie and digitised with an ImageScanner III (GE Healthcare).

### In-Gel Trypsin Digestion and LC MS/MS

Individual gel regions corresponding to major protein bands from different polyacrylamide gels were manually excised, destained, reduced, carbamidomethylated, and trypsin digested as described in Addis *et al*.^41^. Tryptic peptides were supplied to the proteomic facility of Porto Conte Ricerche Srl (Tramariglio, Alghero, Italy) for LC MS/MS analysis using a XCT Ultra 6340 ion trap equipped with a 1200 HPLC system and a chip cube (Agilent Technologies, Palo Alto, CA) as described in Biosa *et al*.^42^. Mass spectrometry output data were analysed on the software provided by the manufacturer (6300 Series Ion Trap LCMS) employing Mascot Daemon MS/MS ion search software (Version 2.3, Matrix Science, Boston, MA) for protein identification. Data were then processed against the NCBI database (http://www.ncbi.nlm.nih.gov).

### Oligonucleotides, plasmid construction, and sequencing

*B. laterosporus* genomic DNA used in PCR reactions was extracted and purified from overnight cultures employing the Wizard^®^ Genomic DNA Purification Kit (Promega) in compliance with the manufacturer’s instructions.

Oligonucleotides including the *Bam*HI and *Eco*RI recognition site (used to digest also pGEX-3X vector) were synthesised (Life Technologies) and employed for PCR amplification of *exsC, chrd, cpbA* and *cpbB* genes. These primers were designed on sequences of homologous genes previously reported in GenBank (Table 1).

PCR reactions were performed in a volume of 25 μl per sample employing *Pfu*^®^ DNA Polymerase in compliance with manufacturers’ instructions (Promega^®^, Madison, USA). with the following thermal profile: 95 °C for 5 min, 30 cycles at 95 °C for 30 s, 50–55 °C (50 °C for *exsC* and *chrd;* 55 °C for *cpbA* and *cpbB*) for 30 s and 72 °C for 30 s, and an additional polymerisation step at 72 °C for 10 min.

PCR products were analysed by 1% agarose gel electrophoresis using SYBR^®^ Safe DNA stain (Life Technologies Europe BV, Bleiswijk, The Netherlands) for DNA visualisation.DNA fragments were ligated into pGEX-3X vector (GE healthcare) following manufacturer’s protocol^43^,^44^. Following digestion with *Bam*HI and *Eco*RI enzymes (New England Biolabs), ligation reactions were conducted with T4 DNA ligase (Promega) in compliance with manufacturer’s recommendations. Ligation products were then transformed into chemically competent *E. coli* DH5α by heat shock^45^. Plasmid DNA was isolated from *E. coli* using Wizard^®^ *Plus* SV Minipreps DNA Purification System (Promega) and supplied to the Sanger sequencing facilities of BMR Genomics (Padova, Italy) for sequencing using the pGEX 5′ and 3′ standard primers 5′GGGCTGGCAAGCCACGTTTGGTG3′ and 5′CCGGGAGCTGCATGTGTCAGAGG3′.

### Induction, expression and purification of fusion proteins

GST-tagged recombinant proteins were expressed in *E. coli* BL21 transformed with pGEX-3X fusion constructs. For each protein, an aliquot of an overnight pre-culture was used to inoculate a fresh complex medium (LB) containing 50 μg/ml Ampicillin (Serva) and maintained at 37 °C up to mid-log phase (A_600_ = 0.4–0.6). Cultures were then incubated at 30 °C for 3 h with IPTG (1 mM) for induction. Bacterial cells were harvested and washed twice in cold PBS, before being resuspended in PBS (replaced by distilled water when samples were prepared for insect bioassays) and added with 1 ml acid-washed glass beads (710–1180 μm) (Sigma-Aldrich) for cell breaking through 5 cycles of vortexing (20 s at 100 W) and cooling in ice (20 s). Lysates were then centrifuged at 5,000 rpm for 30 min at 4 °C, and the resulting pellets and supernatants were stored at −20 °C for further use, after being quantified for protein content by Bio-rad Protein assay (Bio-rad).

GST-tagged proteins were purified from lysates following the Glutathione Sepharose 4 Fast Flow one-step protocol (GE Healthcare) according to manufacturer’s instructions, which allowed to collect different protein eluates.

The result of each expression and purification step was checked by SDS-PAGE or Western Blot analysis.

### Western blot

Protein samples were mixed with protein Laemmli buffer, heated at 95 °C for 5 minutes, and resolved by standard SDS-polyacrylamide gel electrophoresis (SDS-PAGE) in order to be transferred to Immobilion-P transfer membranes (Millipore). After being blocked with 3% semi skimmed milk in PBS, the membranes were incubated for 16 h in the same buffer containing Tween 20 (0.05%) and anti-GST polyclonal antibody (GE Healthcare Life Sciences), at 4 °C. Subsequently, the membranes were washed and incubated with anti-goat secondary antibody (peroxidase-conjugated, Sigma-Aldrich) for 1 hr at room temperature. After membrane washing, proteins were visualised by Clarity Western ECL Substrate (Bio-Rad) in VersaDoc MP4000 (Bio-Rad), and the densitometric analysis was performed using Quantity One software.

### Quantitative real-time RT-PCR analysis (RT-qPCR)

The relative expression levels of *exsC, chrd, cpbA* and *cpbB* mRNAs in different bacterial stages of growth were examined by quantitative real-time PCR (RT-qPCR). For this purpose, aliquots of synchronised cultures were harvested at consecutive time intervals (12, 18, 24 h), corresponding to initial stationary (old vegetative cells), early sporulation (initial spore formation) and late sporulation (full spore formation) phases, respectively. The bacterial samples, including three biological replicates, were immediately resuspended in TRIzol^®^Reagent (Life Technologies) before being subjected to 12 cycles (30 s) of sonication (100 W, 40 kHz) and cooling in ice. Then, RNA extraction procedures followed the protocol of Chomczynski and Sacchi^46^. Extracted RNA (1 μg), treated with RQ1 RNase-Free DNase (Promega), was reverse transcribed to complementary DNA (cDNA) with SuperScript^®^ II Reverse Transcriptase and RNaseOUT™ Recombinant Ribonuclease Inhibitor, using a mix of random hexamer primers, according to manufacturer’s instructions (Life Technologies). Quantitative PCR experiments were carried out soon after the synthesis of cDNA. Reactions were conducted using *Power* SYBR^®^ Green PCR Master Mix and were run on an Applied Biosystems 7900HT Fast Real-Time PCR System according to manufacturer’s instructions (Life Technologies) with the following cycle conditions: denaturation at 95 °C for 10 min, followed by 40 cycles of 95 °C for 15 s, annealing at 60 °C for 1 min, and extension at 60 °C for 1 min. The forward and reverse primers of the genes analysed are shown in Table 1. Primers efficiency was preliminarily tested by standard curve and dissociation curve analyses^47^. Each sample was run in technical triplicates and *16S rRNA* was used as *B. laterosporus* internal control gene for PCR normalisation. The relative abundance of qPCR transcripts was calculated in agreement with Livak and Schmittgen^48^.

### Insect bioassays

The bioinsecticidal action of different bacterial preparations was assessed through a standardised bioassay model using newly emerged adults of the house fly *Musca domestica*, provided by the laboratory rearing facility of the Dipartimento di Agraria of the University of Sassari (Italy)^15^. All experiments were carried out in a bioassay room at 25 °C and a photoperiod of L14:D10. The experimental design involved four replicated groups of 10 flies maintained in plastic cages (10 × 15 × 5 cm) and fed daily with 75 μl saccharose solution (30%) containing the bacterial preparation, administered through three capillary tubes (25 μl each). An untreated control, fed just the saccharose solution (30%), was included in each experiment and mortality was assessed every day for 5 days. The following bacterial preparations, produced as previously described, were assayed in different experiments: (1) pure suspensions of progressive *B. laterosporus* phases of growth at a standard concentration of 10^9^ cells/sporangia/spores per ml; (2) lysates of transformed *E. coli* BL21 cells harvested after inducing the expression of GST-tagged recombinant proteins vs non induced cells (protein concentration: 2 mg/ml); (3) purified fusion proteins at a concentration of 1 mg/ml. All experiments were repeated at least three times with different batches of flies and bacterial cultures.

### Statistical analysis

Statistical analyses were performed with SAS software (version 9.1) with significance level set at α = 0.05^49^.

Data are presented as mean ± SEM. Insect mortality data on the 5^th^ day were analysed by one-way ANOVA followed by Least Significant Difference (LSD) tests for post-hoc comparison of means. P value smaller than 0.05 (*p* < 0.05) was considered as significant difference. qPCR data of time course expression experiment and over time mortality data of insect bioassays were analysed using repeated measures ANOVA (PROC MIXED), and means were separated using LSMEANS comparison (adjust = Turkey).

## Additional Information

**How to cite this article**: Marche, M. G. *et al*. Spore surface proteins of *Brevibacillus laterosporus* are involved in insect pathogenesis. *Sci. Rep.* **7**, 43805; doi: 10.1038/srep43805 (2017).

**Publisher's note:** Springer Nature remains neutral with regard to jurisdictional claims in published maps and institutional affiliations.

## Figures and Tables

**Table 1:** Primer sequences used for cloning and qPCR.

Gene	Experiment	Primer sequence^a^	
		Sense 5′- 3′	Antisense 5′- 3′
*chrd*	Cloning	AAGGATCCCCATGACAATACAATTC	CGGAATTCCTAGCATTTACAAATACAA
*exsC*	Cloning	AAGGATCCCCATGGTACGTATTC	GGGGAATTCTTAAGCCTCGTC
*cpbA*	Cloning	AAGGATCCGGATGAAGAGGTTGAAAA	CCGGAATTCTTATTTAAATTGATCTGC
*cpbB*	Cloning	AAGGATCCCCATGAAAATTAGCCCTC	CGGAATTCCTATTTACACGGGCGTAC
*chrd*	qPCR	GGAAGTCCCACCTGTTCTCA	CGGCCCATTAACACCTCTAA
*exsC*	qPCR	TGATCGTTTCGACGTAGCAC	TTAGTAATGCCCCGAACACC
*cpbA*	qPCR	GCTTCACACGATCAGCAACC	TGTAGGCGGGCAGCTAAAAA
*cpbB*	qPCR	TCACCAAGACACAAAGCCCT	GGGCTTTGTGTCTTGGTGAG
*16S rRNA*	qPCR	TGTAGCGGTGAAATGCGTAG	GCGGCACTAAGGGTATTGAA

^a^Underscored the *Bam*HI and *Eco*RI recognition sites.
